# Convolutional neural network advances in demosaicing for fluorescent cancer imaging with color–near-infrared sensors

**DOI:** 10.1117/1.JBO.29.7.076005

**Published:** 2024-07-23

**Authors:** Yifei Jin, Borislav Kondov, Goran Kondov, Sunil Singhal, Shuming Nie, Viktor Gruev

**Affiliations:** a University of Illinois at Urbana-Champaign, Department of Electrical and Computer Engineering, Urbana, Illinois, United States; b Ss. Cyril and Methodius University of Skopje, Department of Thoracic and Vascular Surgery, Skopje, North Macedonia; c University of Pennsylvania, Perelman School of Medicine, Department of Thoracic Surgery, Philadelphia, Pennsylvania, United States; d University of Illinois at Urbana-Champaign, Department of Bioengineering, Urbana, Illinois, United States; e University of Illinois at Urbana-Champaign, Beckman Institute for Advanced Science and Technology, Urbana, Illinois, United States; f University of Illinois at Urbana-Champaign, Carle Illinois College of Medicine, Urbana, Illinois, United States

**Keywords:** near-infrared imaging, image-guided surgery, cancer surgery, bioinspired sensors, demosaicing, convolutional neural network

## Abstract

**Significance:**

Single-chip imaging devices featuring vertically stacked photodiodes and pixelated spectral filters are advancing multi-dye imaging methods for cancer surgeries, though this innovation comes with a compromise in spatial resolution. To mitigate this drawback, we developed a deep convolutional neural network (CNN) aimed at demosaicing the color and near-infrared (NIR) channels, with its performance validated on both pre-clinical and clinical datasets.

**Aim:**

We introduce an optimized deep CNN designed for demosaicing both color and NIR images obtained using a hexachromatic imaging sensor.

**Approach:**

A residual CNN was fine-tuned and trained on a dataset of color images and subsequently assessed on a series of dual-channel, color, and NIR images to demonstrate its enhanced performance compared with traditional bilinear interpolation.

**Results:**

Our optimized CNN for demosaicing color and NIR images achieves a reduction in the mean square error by 37% for color and 40% for NIR, respectively, and enhances the structural dissimilarity index by 37% across both imaging modalities in pre-clinical data. In clinical datasets, the network improves the mean square error by 35% in color images and 42% in NIR images while enhancing the structural dissimilarity index by 39% in both imaging modalities.

**Conclusions:**

We showcase enhancements in image resolution for both color and NIR modalities through the use of an optimized CNN tailored for a hexachromatic image sensor. With the ongoing advancements in graphics card computational power, our approach delivers significant improvements in resolution that are feasible for real-time execution in surgical environments.

## 1 Introduction

The prevalence of cancer, with one in every three individuals globally being affected, highlights the indispensable role of surgical procedures in addressing localized cancers.^1^^,^^2^ The success of these interventions, pivotal for enhancing patient survival rates, largely depends on the thorough removal of primary tumors and the prompt detection of any metastatic occurrences.^3^^–^^5^ Advancements in near-infrared (NIR) image-guided surgery are at the forefront of improving surgical accuracy and patient prognosis.^6^ This innovative approach leverages cutting-edge tumor-targeted probes,^7^^,^^8^ state-of-the-art imaging devices,^9^ and the integration of machine learning algorithms into surgical workflows,^10^ significantly aiding surgeons in identifying primary tumors with unprecedented precision.^11^^,^^12^ The recent regulatory approval of CYTALUX, a folate-targeted probe for lung and ovarian cancers, and Lumicell, a cathepsin-activated probe for breast cancer, marks a milestone in utilizing tumor-targeted probes within the NIR spectrum.^13^^–^^15^ In addition, several promising tumor-targeted agents are on the cusp of completing phase III clinical trials, poised to soon make a significant impact on the market and further revolutionize the field of NIR image-guided surgery.^16^

The journey of intraoperative imaging instruments for NIR image-guided surgery spans over a decade, paralleling advancements in compact sensor technologies, enhanced quantum efficiency, and sensitivity within the NIR spectrum. This evolution mirrors the historical progression of color imaging technologies.^17^^,^^18^ Initially, color imaging relied on time multiplexing technology, utilizing rotating spectral filters (red, green, and blue) in front of a sensor, assuming a static scene during the acquisition of each color. The challenges of motion artifacts introduced by this technique were later mitigated by incorporating a beam splitter in the optical path, dividing the incoming light into three channels to simultaneously capture color images, thereby enabling real-time, high-resolution video capture.^9^^,^^19^ However, this solution increased the imaging sensor’s size, rendering it impractical for numerous applications. Temperature-dependent coregistration further complicates matters.^20^ The advent of Bayer filters revolutionized color imaging,^21^ leading to the single-chip color sensor becoming the predominant technology over the past five decades. The demand for compact color sensors, driven largely by the mobile phone industry, has fortuitously benefited the endoscopic medical field, fostering new endoscopic applications.

NIR imaging instruments have traced a similar trajectory.^22^^–^^25^ Initial models employed a single NIR filter over a grayscale imaging device to capture NIR fluorescent images. This approach was expanded through time-multiplexing color and NIR filters, facilitating the simultaneous imaging of visible and NIR fluorescence.^26^ However, the same motion artifact challenges observed in color imaging were encountered, leading to the adoption of a beam splitter solution.^27^ This method, dividing the incoming light into two paths for color and NIR fluorescence imaging, has become prevalent among food and drug administration-approved instruments. Yet, as the focus shifts toward minimally invasive techniques^28^ and imaging multiple NIR fluorescent probes, this approach is showing its limitations.

Drawing inspiration from the 1975 introduction of the Bayer color filter array, our group has developed pixelated color–NIR filters optimized for NIR fluorescent imaging and seamlessly integrated with an imaging device.^20^^,^^29^^,^^30^ This innovation addresses coregistration issues and improves power and data efficiency. To enhance the imaging of multiple NIR fluorescent markers, we combined vertically stacked imaging technology with pixelated spectral filters, enabling the simultaneous capture of color images and three distinct observations in the NIR spectrum. This technology, inspired by the visual system of the mantis shrimp, facilitates the concurrent imaging of two NIR fluorescent probes and three-dimensional (3D) reconstruction using NIR structured illumination.

However, similar to the Bayer filter in color imaging,^21^ pixelated NIR–color cameras experience a reduction in spatial resolution.^31^^,^^32^ Fortunately, this challenge is not new to color imaging, and various demosaicing techniques have been developed to mitigate spatial resolution loss.^33^ Color demosaicing techniques, essential for converting raw sensor data into full-color images, can be broadly classified into three main approaches: interpolation, dictionary-based, and learning-based. Interpolation methods, known for their computational simplicity, leverage the local attributes of an image and the correlation between color channels to estimate missing pixels.^34^ However, this approach often results in zipper artifacts and inaccuracies in color representation. To mitigate some of these drawbacks, adaptive and iterative interpolation methods have been developed, offering improved results at the cost of increased computational demands and slower processing times, hindering real-time application.^35^ Dictionary-based methods, on the other hand, employ interpolation strategies informed by learned image patches,^36^ leading to enhanced spatial detail and more accurate color reproduction. These approaches draw from a predefined set of patterns to better reconstruct the image, balancing efficiency with improved image quality.

Convolutional neural networks (CNNs) have emerged as a powerful tool in addressing both low- and high-level challenges in computer vision, spanning tasks from image enhancement and object recognition to surgical guidance and intraoperative robotics.^37^^,^^38^ Specifically in the realm of image demosaicing, CNNs have outperformed traditional state-of-the-art techniques by mastering the direct translation from mosaic images to their full-resolution counterparts.^39^ This is achieved through the network’s exposure to vast datasets of mosaic and corresponding full-resolution images, enabling it to discern and learn vital features within the local vicinity of an image, such as edges, noise patterns, inter-channel correlations, and other nuanced details critical for accurately filling in missing color information. Initially conceived for image super-resolution,^40^ this approach was successfully adapted for color demosaicing, applying to image sensors equipped with Bayer filters or similar color filter arrays, showcasing the versatility and efficacy of CNNs in modern image processing tasks.^39^

**Fig. 1:**
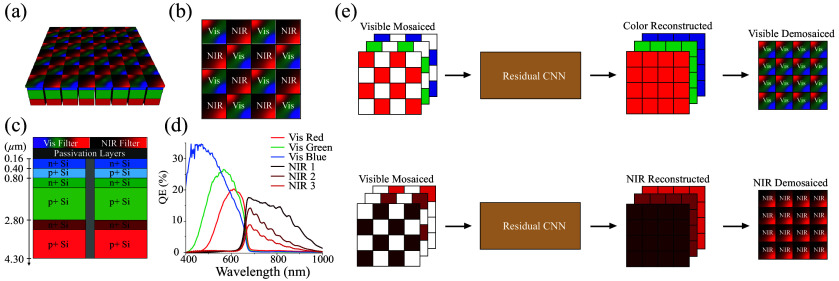
Schematic overview of our bioinspired sensor with spectral sensitivity and a residual CNN for demosaicing color and NIR images: panels (a)–(c) depict the configuration of our hexachromatic image sensor, incorporating pixelated filters and vertically stacked photodiodes. Panel (d) details the quantum efficiency of our bioinspired sensor. Panel (e) illustrates the design of the residual CNN, featuring two separate networks for demosaicing the color and NIR channels.

In this paper, we introduce a CNN-based approach for demosaicing images captured with a hexachromatic imaging sensor (Fig. 1). This sensor incorporates two distinct types of pixelated spectral filters arranged in a checkerboard pattern: one set designed to transmit visible light wavelengths from 400 to 700 nm and another set tailored to permit NIR light wavelengths from 700 to 1000 nm. Given the pixelated design of the sensor, the spatial resolution in both the visible and NIR spectra is effectively halved. To address this, we developed a CNN model specifically trained to restore the diminished spatial resolution inherent to our sensor’s unique architecture. The performance of our CNN-based demosaicing method is evaluated against traditional interpolation techniques using a benchmark dataset (referred to as the Waterloo image dataset) and a selection of images captured with a vertically stacked image sensor. In addition, we present a series of pre-clinical and clinical images to demonstrate the practical application and effectiveness of our approach.

## 2 Deep Learning-Based Demosaicing Implementation

**Fig. 2:**
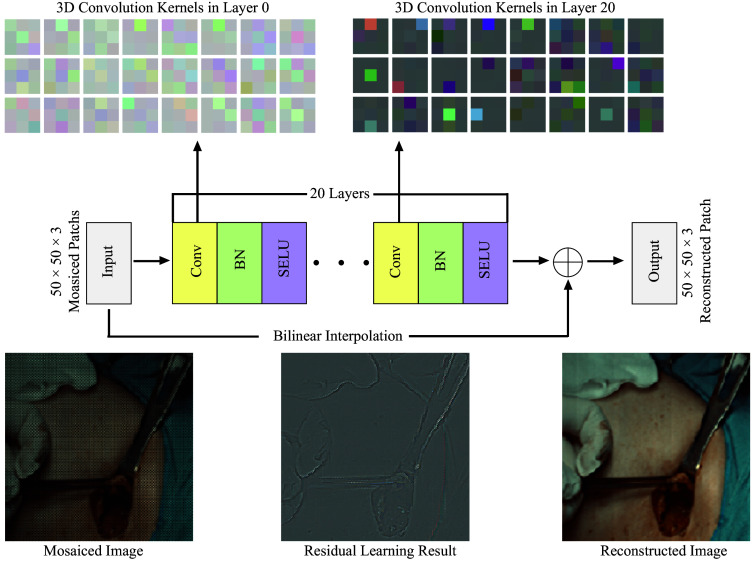
Our deep residual CNN is constructed with 20 layers, each layer consisting of a convolutional segment that utilizes 64 filters with 3 × 3 receptive fields, complemented by batch normalization and a SELU activation function. This network is adept at learning residuals, which are subsequently integrated with a bilinearly interpolated image to produce the final demosaiced image. The model incorporates 3D convolution kernels in both the initial and concluding layers, and we provide illustrations of both the intermediate results from the CNN and the ultimate image.

In this work, we utilized CNN tailored specifically for the task of demosaicing color and NIR images captured by our novel bioinspired hexachromatic imaging sensor. Drawing upon the foundational principles of deep residual learning^41^ and leveraging insights from super-resolution techniques^40^ and CNN-based demosaicing research,^39^ we optimized a very deep CNN within this framework. The architectural blueprint of our CNN demosaicing model is depicted in Fig. 2, showcasing a structure designed to overcome the common pitfalls associated with deep network training.

Deep CNNs, characterized by their numerous layers, operate under the premise that increased depth should theoretically yield higher accuracy in tasks such as image demosaicing. However, empirical observations reveal a paradox where, beyond a certain depth, the model’s performance plateaus and may even deteriorate, an issue not attributable to overfitting.^42^ This degradation in performance with increased depth highlights challenges in network optimization rather than model complexity per se.

To address the challenges of optimizing deep learning models for image demosaicing, we integrate the residual learning framework into our strategy, leveraging its proven success in the fields of super-resolution and color image demosaicing.^39^^,^^40^ The essence of this approach is its focus on high-frequency details, regarded as residuals, which the network learns to predict directly from the data, rather than reconstructing the entire image anew. This method is especially suited to our application, given the separate spectral information provided by the color and NIR channels and their independent needs for interpolation.

The process begins with both color and NIR images at a resolution that is half of what the sensor is capable of capturing (Fig. 2). To accurately mirror the pixelated filter pattern on the image sensor, the initial pixel of both the color and NIR images is offset by one in the horizontal direction. Initially, missing pixels are estimated using bilinear interpolation, a method chosen for its computational efficiency despite its tendency to introduce zipper artifacts and color inaccuracies. However, these drawbacks are somewhat mitigated in our hexachromatic imager compared with traditional Bayer color filter arrays. The interpolated image serves as the input for the CNN, which is tasked with refining the accuracy of the pixel estimates to achieve full resolution. Importantly, this bilinearly interpolated image retains the low-frequency content of the eventual full-resolution output, given that half of the pixels in both images are identical. Thus, the CNN is specifically trained to discern and learn the high-frequency details that differ, facilitating a more focused and efficient learning process.

This approach not only streamlines the model’s training by emphasizing the learning of high-frequency information over the entire image but also enhances the depth and learning rate capabilities of the CNN. Consequently, the final full-resolution image emerges from the combination of the bilinear interpolated base and the high-frequency residuals refined by CNN.

Our CNN architecture consists of 20 layers, each incorporating a convolutional layer, a batch normalization, and a scaled exponential linear unit (SELU) activation function. Every convolutional layer employs 64 filters with 3 × 3 receptive fields and one-pixel padding to maintain the spatial dimensions of the input, except for the final layer, which uses three filters also with 3×3 receptive fields, tailored for the demosaicing output. The examples of the receptive fields from both the first and last layers are displayed in Fig. 2. The mathematical framework for this demosaicing process is as follows:

$$F_{n}(Y)=\mathrm{selu}(Y*Wn+Bn),\;n=1\ldots N-1,$$ (1)

$$\mathrm{SELU}(x)=\{\begin{array}{ll} \lambda x & \text{if }x>0\\ \lambda \alpha (e^{x}-1) & \text{if }x\leq 0, \end{array}$$ (2)

F(Y)=FN−1(Y)*WN+BN. (3)

In Eq. (1), Y is the input patch; $F_{n}$ is the output feature map of the n’th layer; $W_{n}$ and $B_{n}$ represent the filters and the bias vector of the n’th layer, respectively; and * is the convolution operator. This equation represents a convolution operation and the extraction of useful local features in the image. To maintain the spatial dimensions of the input, we utilized 64 filters with 3×3 receptive fields and one-pixel padding in all layers except the final one. For the final layer [Eq. (3)], we used three filters with 3×3 receptive fields, specifically designed for the demosaicing output. Each layer consists of a convolutional layer, followed by a batch normalization layer, and a SELU activation function. Furthermore, λ and α are constants as defined in the SELU activation function literature. The optimization of the network utilizes a modified L2-norm for the loss function

L(Θ)=1n∑i=1n‖(F(Yi;Θ)+Y^i)−Xi‖2, (4)

where $F(Y_{i};\theta )$ represents the predicted high-frequency components or residuals that, when added to the bilinearly interpolated image $Y_{i}$, approximate the ground truth high-resolution image $X_{i}$. The network initialization follows the MSRA policy, and the Adam optimizer is employed for gradient updates with a learning rate set to $1\times 10^{-5}$. This setup ensures that the network effectively learns the residuals necessary for reconstructing the high-resolution image from its lower-resolution counterpart.

In our study, we utilized a dataset comprising 4744 images from the Waterloo Exploration Dataset (WED).^43^ This dataset was chosen for its diverse range of color scenes and intricate high-frequency patterns, offering the true color values at every pixel. Such variability in color and spatial detail was instrumental in training the CNN to identify features under varying conditions and accurately predict the missing pixel values. From each image, we extracted 100 patches of 50×50  pixels to serve as the training data for the network. The rationale for selecting this patch size is grounded in the nature of demosaicing as a process that predicts missing pixel values based on nearby information; thus, local context is more critical for this task than global context, which might be more important for other computer vision tasks such as object recognition. To enhance our dataset further, we applied data augmentation techniques to each patch by rotating them four times at 90-deg intervals and applying horizontal flips, thereby enriching the training data and improving the robustness of the network. Of the WED images, 4644 are used for training purposes, and 100 are used for testing.

To determine the optimal number of layers for the residual CNN model, we trained models with 5, 10, and 15 layers using the WED dataset. The average peak signal-to-noise ratio (PSNR) for image reconstruction on 100 WED test images was 33.5 dB for the 5-layer model, 33.8 dB for the 10-layer model, and 34.5 dB for the 15-layer model. All of these performances were lower than the 20-layer model, which achieved a PSNR of 35.81 dB. Given the balance between training resources and model complexity, the 20-layer model is considered optimal.

The architecture of our CNN is designed to process both color and NIR images with the same underlying structure. Given the minimal spectral overlap between the color and NIR spectrums, we posit that the interrelation between these pixel sets is negligible, allowing for their independent analysis. We anticipate that the NIR channels will display high-frequency structures akin to those found in color images. An example of this is seen with NIR fluorescence emitted by indocyanine green, which not only illuminates lymph nodes but also outlines the lymphatic vessels.^29^ These vessels, often only a few pixels in width, significantly contribute to the high-frequency detail in the images. Therefore, it is reasonable to expect that NIR images will contain high-frequency information comparable to that of color images. The principal distinction lies in the spectral sensitivity, which differs markedly between our NIR and visible channels in comparison with the Bayer color imaging sensor. Addressing this disparity to optimize the six-channel spectral estimation for full-resolution image reconstruction is a goal for future development.

## 3 Evaluation of Residual CNNs for Demosaicing

**Fig. 3:**
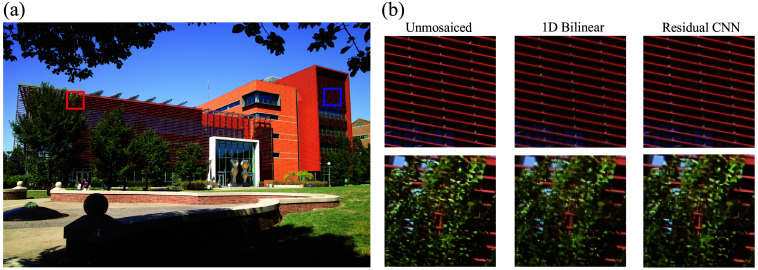
Visual comparison of demosaiced images obtained through bilinear interpolation and the residual CNN framework reveals significant differences. Close-up views highlight how the CNN method successfully avoids zig-zag and false color artifacts, which are prominently visible in images processed by bilinear interpolation.

We evaluate the performance of our demosaicing algorithm using several datasets: Waterloo color images captured with a Bayer color filter array; color images obtained with the Foveon X3 sensor (Fig. 3); the “UIUC Color NIR X3” dataset consisting of color and NIR scenes from the University of Illinois campus captured with vertically stacked photodiodes; the “UIUC NIR Preclinical” dataset featuring animal models of breast cancer imaged with vertically stacked photodiodes and tagged with NIR fluorescent probes; and the “UIUC NIR Clinical” dataset, which includes clinical images from patients with lung or breast cancer, also tagged with NIR fluorescent probes and captured with vertically stacked photodiodes.

The UIUC Color NIR X3 and UIUC NIR Preclinical datasets provide full-resolution images across six spectral channels per pixel: three in the visible spectrum and three in the NIR spectrum. The datasets were captured using a bare image sensor equipped with vertically stacked detectors, omitting any pixelated filter array. Two sets of images were acquired: one using a low-pass filter that blocks all NIR wavelengths above 700 nm and another with a high-pass filter that blocks the transmission of visible photons below 700 nm. This imaging approach simulates the spectral sensitivity profile of our pixelated hexachromatic image sensor. Conversely, the UIUC NIR Clinical dataset lacks ground truth data, containing only pixels with either visible or NIR information as captured by our bioinspired sensor. Given the critical importance of this dataset for assessing our sensor’s clinical performance, we developed an accuracy metric inspired by analogous methodologies in the color imaging domain. Finally, the Waterloo dataset is used to evaluate the demosaicing algorithms on color data collected with a Bayer color sensor. Because we use 90% of this dataset to train our CNN model, we use the remaining 10% to evaluate its demosaicing results. The downside of this evaluation is that the difference in spectral response between the two cameras can provide slight biases in the CNN toward Bayer-type sensors.

### 3.1 Color Demosaicing on Images Obtained with Foveon-Sigma Camera

To evaluate the performance of our CNN-based demosaicing routine on images with diverse colors and textures, we utilized an unmosaiced reference image taken by a commercial camera (DP1x, Sigma, Marietta, Georgia, United States) without a color filter array. A short-pass filter, allowing only the visible spectrum, was attached atop the camera to capture a still image of 2640×1760  pixels. This reference image, depicted in Fig. 3(a), showcases the Electrical and Computer Engineering Building at the University of Illinois at Urbana-Champaign.

We then applied a color–NIR filter pattern to the reference image to simulate the visible pixels captured by a hexachromatic camera. This mosaiced color image served as the input to our model. To assess our model’s accuracy, we compared the demosaiced images with the original unmosaiced reference image, treating it as the ground truth for evaluating the image reconstruction quality. As a comparative baseline, a one-dimensional (1D) bilinear interpolation was implemented alongside our model.

In Fig. 3(b), two 200×200 patches, one of a canopy and the other of a façade, illustrate the comparison. The bilinear demosaicing of the canopy exhibits numerous artificial artifacts on the leaves and visible mosaicing, whereas our model significantly reduces these imperfections, resulting in a reconstruction closer to the ground truth. Similarly, for the façade, our model produces smoother reconstructions of high-contrast elements, such as wires, outperforming the rougher appearance generated by bilinear interpolation.

For the quantitative evaluation of the demosaicing performance, we employed four metrics: PSNR, mean squared error (MSE), the 95th percentile of the structural dissimilarity index measure (DSSIM), and the 95th percentile color difference (ΔE). Our CNN model showed substantial improvements across these metrics compared with bilinear interpolation, with increases in PSNR across the color channels and significant reductions in MSE, indicating overall enhanced image quality. Specifically, the PSNR improved by an average of 2.25 dB, and the MSE saw a 40.6% reduction (see Table 1). Using an independent two-sample t test on the residuals of CNN and 1D bilinear interpolation with 100 images from the Waterloo dataset, we obtained p values of 0.00027, 0.00003, and 0.00004 for the three color channels. These results indicate a statistically significant difference in PSNR between the two methods.

**Table 1:** Effectiveness of our residual CNN compared with bilinear interpolation using the Waterloo dataset, along with full images and sub-images from the Foveon X3, which demonstrates that the residual CNN consistently surpasses the bilinear demosaicing method in all evaluated metrics and datasets.

Metric	Waterloo 100		Foveon X3 full res		Foveon X3 canopy		Foveon X3 façade	
	1D bilinear	Residual CNN	1D bilinear	Residual CNN	1D bilinear	Residual CNN	1D bilinear	Residual CNN
PSNR–Vis R (dB)	33.22 ± 3.57	35.97 ± 3.70	29.14	31.39	24.80	26.13	26.38	34.09
PSNR–Vis G (dB)	33.06 ± 3.05	35.81 ± 3.28	29.12	31.32	24.76	26.12	28.66	35.78
PSNR–Vis B (dB)	32.99 ± 2.98	35.67 ± 3.28	29.64	31.98	25.45	26.90	29.75	35.79
MSE–Vis R	0.387 ± 0.316	0.215 ± 0.197	0.660	0.393	2.212	1.626	1.471	0.249
MSE–Vis G	0.360 ± 0.281	0.201 ± 0.194	0.706	0.425	2.250	1.644	1.689	0.327
MSE–Vis B	0.402 ± 0.390	0.234 ± 0.300	0.663	0.387	2.499	1.790	1.601	0.399
% ΔE	4.203 ± 1.933	3.120 ± 1.509	7.328	5.076	13.072	11.483	10.349	3.988
% DSSIM	0.138 ± 0.060	0.096 ± 0.058	0.172	0.140	0.216	0.179	0.068	0.025

Focusing on the reconstruction quality of smaller image patches with detailed colors and textures, we analyzed two 200 × 200 pixel patches: one of the building façade and one of the canopy. For these detailed sections, our model still outperformed the baseline but with slightly reduced margins compared with the full-image analysis. Nevertheless, the performance in reconstructing high-contrast details, such as the building façade, was particularly noteworthy, with our model achieving significantly better PSNR, a 79.5% reduction in MSE, and marked improvements in DSSIM and ΔE, underscoring our model’s superior capability in handling images with high-contrast patterns (see Table 1). Comparable improvements in image quality were also noted on the Waterloo image dataset, as detailed in Table 1.

### 3.2 Color and NIR Demosaicing on Hexachromatic Images

To broaden the scope of our model to encompass multi-spectrum imaging, we assessed our demosaicing approach using 20 images from the UIUC Color NIR X3 dataset, published by Blair and Gruev.^31^ These images were captured with a custom camera equipped with both short-pass and long-pass filters, selectively blocking the NIR and visible spectrums, respectively. This setup, featuring a sensor with three vertically stacked photodiodes per pixel, enables the generation of unmosaiced ground truth images in both the visible and NIR spectrums by alternating the filters at a 700 nm threshold.

Our CNN model significantly outperforms bilinear interpolation in reconstructing images across the visible spectrum. Specifically, the model achieves a PSNR increase of 2.41, 2.50, and 2.50 dB for the blue, green, and red channels, respectively, leading to an average PSNR improvement of 2.47 dB for the visible channels. The model also reduces the MSE by 41.4% compared with the baseline. In terms of similarity, the 95% DSSIM is improved by 24.0% with our model. In addition, the color difference across the three channels, measured by 95% ΔE, shows a 14.2% improvement with our model (see Table 2).

**Table 2:** Our residual CNN against bilinear interpolation across various datasets, including UIUC Color NIR X3, UIUC NIR Preclinical, and UIUC NIR Clinical images for breast and lung cancer, which clearly shows that the CNN method outperforms bilinear demosaicing across all metrics and datasets consistently.

Metric	UIUC Color NIR X3		UIUC NIR Preclinical		Breast cancer clinical image		Lung cancer clinical image	
	1D bilinear	Residual CNN	1D bilinear	Residual CNN	1D bilinear	Residual CNN	1D bilinear	Residual CNN
PSNR–Vis R (dB)	38.84 ± 3.19	41.25 ± 2.78	37.60 ± 1.24	39.76 ± 1.56	43.95	46.55	38.49	44.06
PSNR–Vis G (dB)	38.89 ± 2.86	41.39 ± 2.84	37.17 ± 1.21	39.41 ± 1.35	44.39	47.16	38.58	43.92
PSNR–Vis B (dB)	39.54 ± 2.75	42.04 ± 3.02	39.49 ± 1.51	41.49 ± 1.82	43.96	45.84	38.18	44.53
MSE–Vis R	0.198 ± 0.166	0.111 ± 0.087	0.179 ± 0.054	0.116 ± 0.049	0.042	0.023	0.120	0.033
MSE–Vis G	0.189 ± 0.146	0.110 ± 0.097	0.183 ± 0.050	0.115 ± 0.045	0.037	0.019	0.112	0.032
MSE–Vis B	0.174 ± 0.145	0.106 ± 0.102	0.145 ± 0.051	0.099 ± 0.047	0.034	0.022	0.136	0.031
% ΔE–Vis	2.698 ± 0.772	2.315 ± 0.469	2.373 ± 0.441	1.979 ± 0.358	1.291	1.173	2.067	1.975
% DSSIM–Vis	0.107 ± 0.029	0.081 ± 0.028	0.102 ± 0.052	0.081 ± 0.041	0.022	0.016	0.052	0.048
PSNR–NIR 1 (dB)	34.04 ± 2.48	35.84 ± 2.56	47.05 ± 0.46	47.83 ± 1.23	51.66	54.86	47.52	48.58
PSNR–NIR 2 (dB)	37.44 ± 3.24	39.11 ± 3.16	54.10 ± 1.87	54.27 ± 1.63	51.61	54.10	51.30	52.01
PSNR–NIR 3 (dB)	40.91 ± 3.46	42.28 ± 3.24	55.43 ± 1.17	55.54 ± 1.71	38.76	42.89	52.84	53.06
MSE–NIR 1	0.360 ± 0.236	0.231 ± 0.140	0.033 ± 0.008	0.020 ± 0.004	0.095	0.073	0.097	0.076
MSE–NIR 2	0.302 ± 0.179	0.201 ± 0.067	0.072 ± 0.025	0.077 ± 0.027	0.089	0.050	0.210	0.178
MSE–NIR 3	0.195 ± 0.106	0.137 ± 0.069	0.112 ± 0.071	0.116 ± 0.075	0.093	0.052	0.077	0.074
% ΔE–NIR	3.506 ± 1.029	2.923 ± 0.796	0.787 ± 0.087	0.785 ± 0.087	0.596	0.378	0.813	0.794
% DSSIM–NIR	0.116 ± 0.029	0.104 ± 0.032	0.039 ± 0.015	0.036 ± 0.017	0.048	0.029	0.032	0.031

For the NIR spectrum images, our model’s performance demonstrates an enhancement, with PSNR increases of 1.80, 1.67, and 1.37 dB across the NIR channels, culminating in an average PSNR gain of 1.61 dB. The MSE is reduced by 33.0%, and the 95% DSSIM sees a 10.8% increase. The color difference improvement in the NIR channels, indicated by 95% ΔE, is 16.6%. These results highlight our model’s superior demosaicing capabilities over bilinear methods for both visible and NIR spectrums (see Table 2). However, the performance on NIR images suggests room for improvement, likely due to the model being primarily trained on visible spectrum images.

### 3.3 Preclinical Evaluation of Residual CNNs for Color and NIR Image Demosaicing

Expanding our model to include pre-clinical animal studies, we evaluated it using a publicly available dataset featuring three pairs of unmosaiced visible and NIR *in vivo* images of female mice with breast tumors (4T1, American Type Culture Collection, Rockville, Maryland, United States), introduced subcutaneously and grown to a diameter of 1 cm. To highlight the breast tumors with NIR fluorescence, each mouse received an injection of IRDye 800CW Maleimide (100  μL at 11.91  μg per mL in phosphate-buffered saline) into the retro-orbital sinus, allowing for tumor accumulation over 24 h. Image collection was performed using a custom camera equipped with three stacked photon detectors and an excitation filter to omit NIR excitation light. Visible images were captured under white light, and NIR images were obtained under infrared illumination (I0785MU6000M4S, Innovative Photonic Solutions, Plainsboro, New Jersey, United States).

**Fig. 4:**
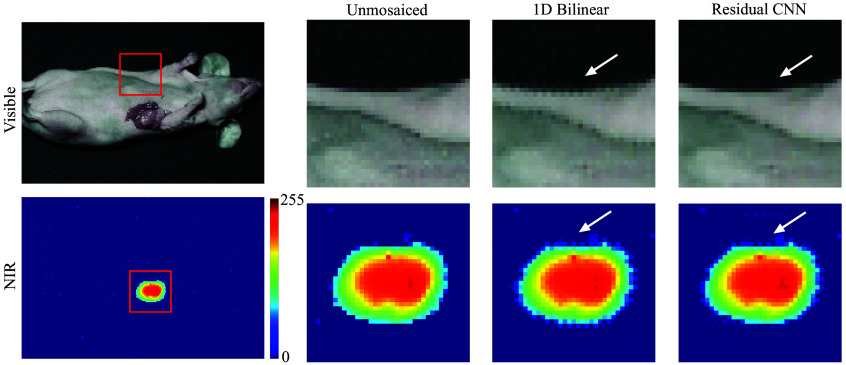
Side-by-side visual evaluation of demosaicing using bilinear interpolation and the residual CNN approach on an animal model with breast cancer illustrates the advantages. The comparison of color and NIR fluorescence images demonstrates how the CNN demosaicing method uncovers superior high-resolution details compared with the bilinear technique.

Applying our model to this dataset yielded notable enhancements across various metrics for both visible and NIR images (see Fig. 4). The PSNR saw increases of 2.15, 2.26, and 2.00 dB across the color channels, with an average PSNR boost of 2.13 dB for the trio. The CNN model’s reconstruction reduced the MSE by 34.7% compared with the baseline. Similarly, the average DSSIM showed a 37.0% improvement, and the color difference (ΔE) was enhanced by 13.6% (see Table 2).

In the NIR spectrum, the PSNR improvement for each channel was 0.78, 0.15, and 0.09 dB due to the low-frequency information of NIR fluorescence and lower sensitivity in the NIR 2 and 3 channels. The model’s MSE reduction in this spectrum was 1.8%, and the 95% DSSIM and NIR color differences show very limited enhancement (see Table 2).

### 3.4 Clinical Evaluation of Residual CNNs for Color and NIR Image Demosaicing

**Fig. 5:**
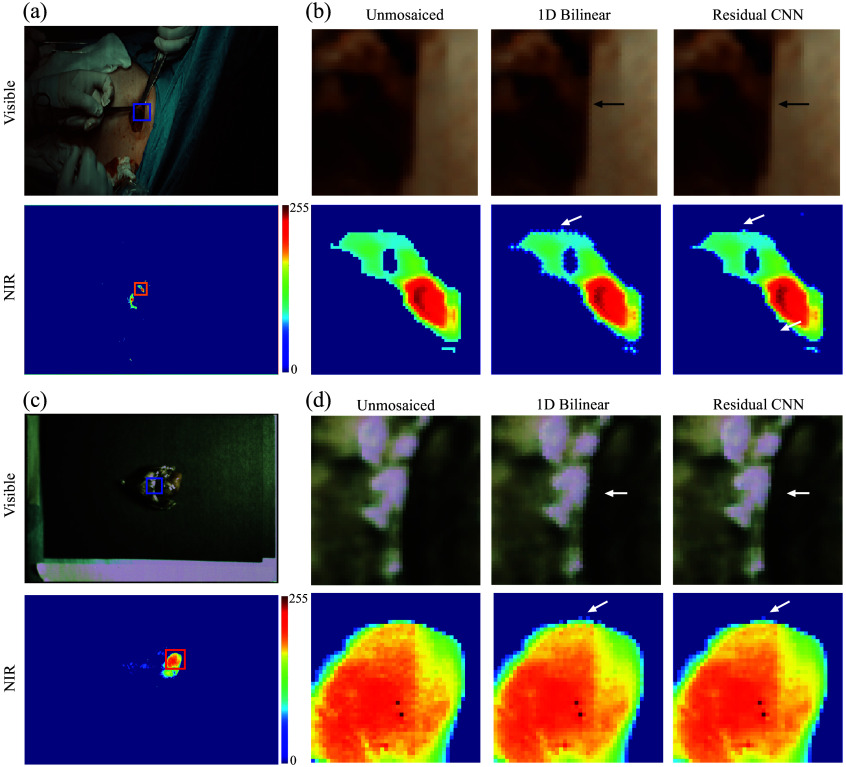
(a), (b) Color and NIR images taken *in vivo* in the operating room from a patient during breast cancer surgery. (c), (d) Color and NIR images taken *ex vivo* on the back table in the operating room from a patient undergoing surgery for lung cancer. The arrows point out the zipper artifacts at the edges in both imaging types when processed through bilinear interpolation, which are significantly reduced by employing our CNN methodology.

Clinical data were acquired using our hexachromatic image sensor in two distinct scenarios (Fig. 5). First, during breast cancer surgery, patients received a peritumoral injection of indocyanine green to delineate the sentinel lymph nodes. The image sensor was mounted above the operation area, enabling the real-time capturing of both color and NIR fluorescence videos as surgeons conducted lymph node mapping. In the second scenario, lung cancer patients undergoing surgical procedures received VGT-309, a cathepsin-targeting indocyanine green (ICG) agent designed to illuminate the tumor microenvironment through fluorescence in areas with heightened cathepsin activity. *Ex vivo* tissues were subsequently imaged directly in the operating room using our hexachromatic sensor, facilitating the detailed observation of tumor-specific fluorescence signals.

**Fig. 6:**
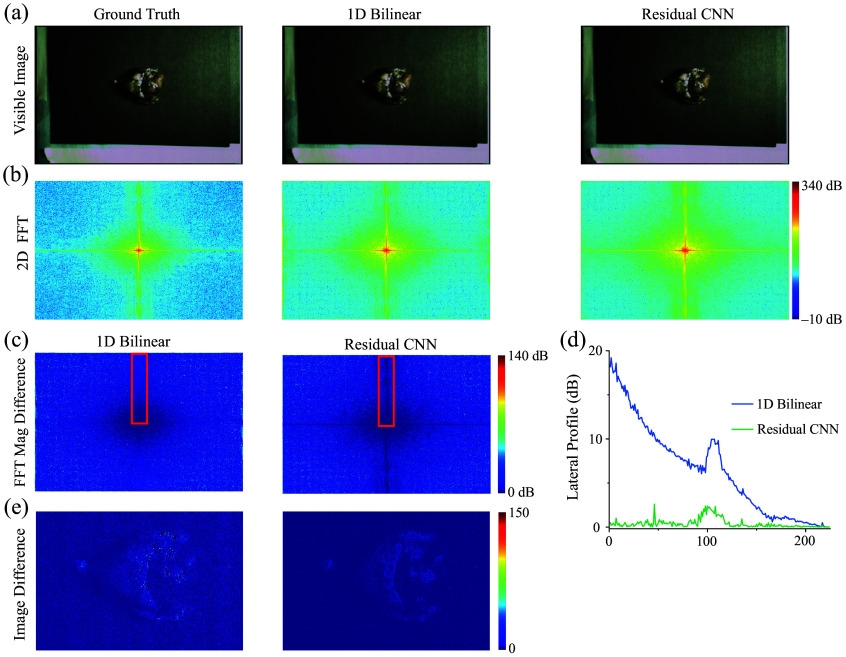
(a) *Ex vivo* color images of lung cancer tissue processed using both bilinear and CNN demosaicing techniques. (b) The magnitude of the Fourier-transformed images reveals that the high-frequency content of the CNN-demosaiced image closely matches that of the original. (c) The comparison between the original and demosaiced images, either by bilinear or CNN methods, further demonstrates the superior spatial reconstruction achieved with the CNN approach. An analysis along the vertical frequencies highlights a 20-dB improvement in the CNN method over bilinear processing at higher frequencies.

Figures 5 and 6 display the original images, those processed by bilinear interpolation, and images enhanced using CNN interpolation. The images enhanced by the CNN display sharper edges and fewer zipper artifacts compared with those processed with bilinear interpolation, as indicated by the arrows on the images in Fig. 5. In addition, Fourier transform analyses reveal variations in the frequency content between the two demosaicing methods relative to the original images (Fig. 6). As anticipated, the bilinear method exhibits a greater discrepancy from the original in terms of high-frequency content due to its inherent limitations. Conversely, the CNN approach, by learning and accurately reconstructing the high-frequency details, significantly reduces errors in these areas of the reconstructed images. This improvement is clearly observable both in the fast Fourier transform (FFT) difference visuals and in the single-line plot across the vertical of the FFT representation. An analysis along the vertical frequencies highlights a 20 dB improvement in the CNN method over bilinear processing at higher frequencies. The images shown in Fig. 6(e) further demonstrate the difference between the original and demosaiced images using bilinear and CNN approaches, respectively. As indicated in the FFT images in Figs. 6(c) and 6(d), the images in Fig. 6(e) show that the CNN method reconstructs the edges more accurately than the bilinear interpolation method.

**Fig. 7:**
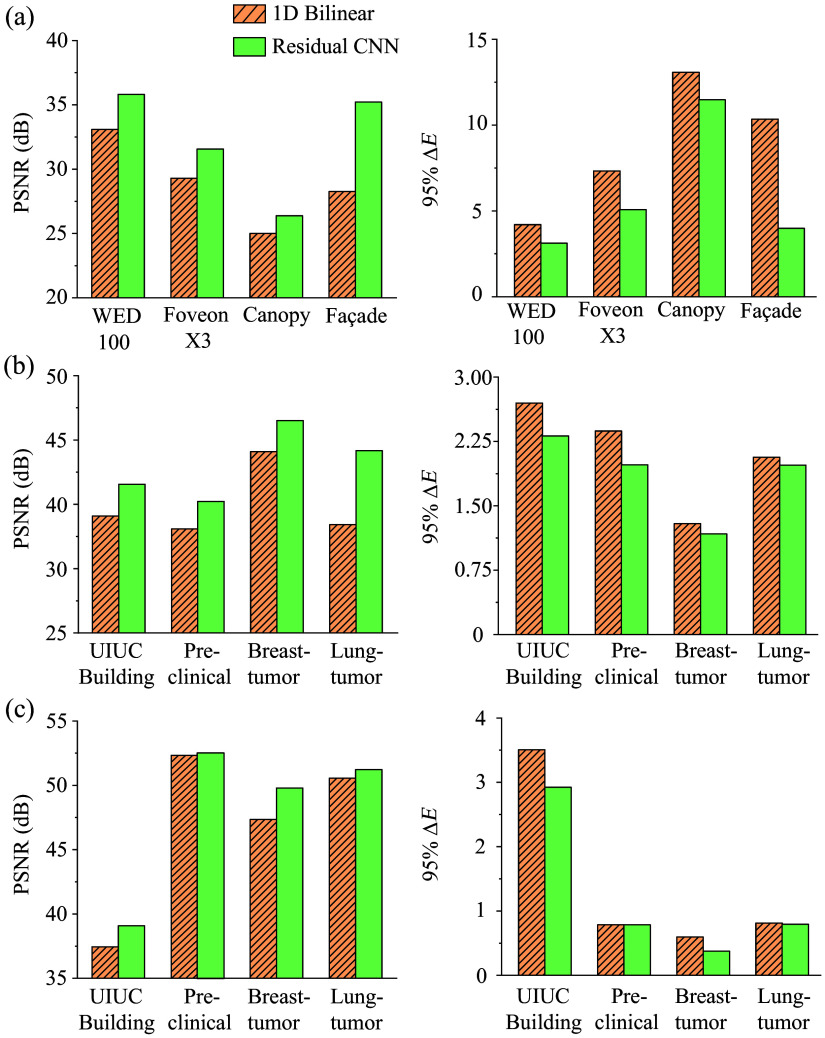
Bar chart comparison of key metrics between bilinear and CNN demosaicing methods across different datasets summarized in (a) Table 1, (b) Table 2 visible channel, and (c) Table 2 NIR channel. Our residual CNN approach consistently outperforms the bilinear demosaicing method across all datasets and metrics.

The data shown in Fig. 6 demonstrate that the CNN method more closely aligns with the original image in terms of FFT magnitude, highlighting its superior performance in image reconstruction. Table 2 and Fig. 7 present a summary of the performance metrics, including PSNR, MMSE, DSSIM, and ΔE, highlighting the CNN’s superior performance over the bilinear method in processing these clinical images.

## 4 Conclusion

In conclusion, our study demonstrates the significant advancements achieved in the field of image demosaicing for medical imaging through the application of a residual CNN. By comparing our CNN model against traditional bilinear interpolation methods across various datasets, including the challenging settings of pre-clinical animal studies and clinical trials, we evidenced marked improvements in image quality metrics, such as PSNR, MSE, DSSIM, and ΔE. Notably, the application of our model to hexachromatic sensor data in the context of fluorescent cancer imaging not only enhanced the clarity and accuracy of both color and NIR images but also minimized high-frequency artifacts, thereby facilitating a more precise identification and analysis of cancerous tissues.

The clinical utility of our hexachromatic image sensor, combined with the CNN-based demosaicing approach, was further underscored in two distinct surgical scenarios: breast cancer lymph node mapping with indocyanine green and lung cancer surgery using a cathepsin-binding ICG agent. These applications highlighted the sensor’s ability to provide real-time, enhanced visualization of tumor environments and sentinel lymph nodes, significantly aiding surgical decisions and outcomes.

Moving forward, the continued refinement and application of CNNs in image demosaicing hold the promise of not only advancing medical imaging technologies but also contributing to more accurate diagnoses, tailored surgical interventions, and ultimately improved patient care. Our findings advocate for the integration of advanced computational methods such as CNNs into the development of next-generation medical imaging devices, aiming to bridge the gap between current limitations and the potential for high-fidelity, multi-spectral imaging in clinical practice.

## Data Availability

The code used to train, validate, and test the neural network was obtained from https://github.com/yifeij7/Residual-CNN and is publicly available.
